# Effects of chiropractic use on medical healthcare utilization and costs in adults with back pain in Ontario, Canada from 2003 to 2018: a population-based cohort study

**DOI:** 10.1186/s12913-023-09690-3

**Published:** 2023-07-25

**Authors:** Jessica J. Wong, Mindy Lu, Pierre Côté, Tristan Watson, Laura C. Rosella

**Affiliations:** 1 Epidemiology Division, Dalla Lana School of Public Health, University of Toronto, 155 College Street, 6th floor, Toronto, ON M5T 3M7 Canada; 2 Institute for Disability and Rehabilitation Research, Ontario Tech University, 2000 Simcoe Street North, Oshawa, ON L1H 7K4 Canada; 3 Faculty of Health Sciences, Ontario Tech University, 2000 Simcoe Street North, Oshawa, ON L1H 7K4 Canada; 4 Institute of Health Policy, Management and Evaluation, University of Toronto, 155 College Street, 4th floor, Toronto, ON M5T 3M7 Canada; 5 ICES, 155 College Street, Toronto, ON M5B 1T8 Canada; 6 Stephen Family Chair in Community Health, Institute for Better Health, Trillium Health Partners, 100 Queensway West, Mississauga, ON L5B 1B8 Canada; 7 Department of Laboratory Medicine and Pathobiology, Temerty Faculty of Medicine, University of Toronto, Medical Sciences Building, 1 King’s College Circle, Toronto, ON M5S 1A8 Canada

**Keywords:** Back pain, Chiropractic, Medical utilization, Costs, Healthcare utilization, Population-based cohort study, Canadian Community Health Survey

## Abstract

**Background:**

Adults with back pain commonly consult chiropractors, but the impact of chiropractic use on medical utilization and costs within the Canadian health system is unclear. We assessed the association between chiropractic utilization and subsequent medical healthcare utilization and costs in a population-based cohort of Ontario adults with back pain.

**Methods:**

We conducted a population-based cohort study that included Ontario adult respondents of the Canadian Community Health Survey (CCHS) with back pain from 2003 to 2010 (n = 29,475), followed up to 2018. The CCHS data were individually-linked to individual-level health administrative data up to 2018. Chiropractic utilization was self-reported consultation with a chiropractor in the past 12 months. We propensity score-matched adults with and without chiropractic utilization, accounting for confounders. We evaluated back pain-specific and all-cause medical utilization and costs at 1- and 5-year follow-up using negative binomial and linear (log-transformed) regression, respectively. We assessed whether sex and prior specialist consultation in the past 12 months were effect modifiers of the association.

**Results:**

There were 6972 matched pairs of CCHS respondents with and without chiropractic utilization. Women with chiropractic utilization had 0.8 times lower rate of cause-specific medical visits at follow-up than those without chiropractic utilization (RR_5years_ = 0.82, 95% CI 0.68-1.00); this association was not found in men (RR_5years_ = 0.96, 95% CI 0.73–1.24). There were no associations between chiropractic utilization and all-cause physician visits, all-cause emergency department visits, all-cause hospitalizations, or costs. Effect modification of the association between chiropractic utilization and cause-specific utilization by prior specialist consultation was found at 1-year but not 5-year follow-up; cause-specific utilization at 1 year was lower in adults without prior specialist consultation only (RR_1year_ = 0.74, 95% CI 0.57–0.97).

**Conclusions:**

Among adults with back pain, chiropractic use is associated with lower rates of back pain-specific utilization in women but not men over a 5-year follow-up period. Findings have implications for guiding allied healthcare delivery in the Ontario health system.

**Supplementary Information:**

The online version contains supplementary material available at 10.1186/s12913-023-09690-3.

## Introduction

Back pain is the leading cause of disability worldwide [1, 2], and a driver of healthcare utilization and costs across health systems [3–9]. One in seven consultations with general practitioners in the United Kingdom were for musculoskeletal conditions, with back pain as the most common reason for care [5]. In Ontario, adults with back pain have 2 times higher rates of back pain-related healthcare visits and 1.2 times higher costs than those without back pain, representing an annual burden of $759 million in Canadian dollars (CAD) [10]. Overall, back pain is associated with high health and economic burden.

Adults commonly consult allied healthcare providers, including chiropractors, for the management of back pain [11, 12]. In Canada, prevalence of chiropractic utilization among adults with back pain was 24% in 2009–2010 [11]. A scoping review of the literature reported that back pain was the most common reason for seeking chiropractic care [12]. Preliminary evidence suggests that use of allied healthcare, including chiropractic care, may be associated with decreased medical visits and costs for spinal conditions [13–15]. Among members of one United States health plan with back pain, patients with chiropractic coverage had fewer surgeries and hospitalizations, and lower back pain-related costs than those without chiropractic coverage [14]. Among adults aged ≥ 65 years enrolled in Medicare, access to chiropractic care was associated with fewer visits to primary care physicians and lower medical spending for spinal conditions [13, 16]. These studies were conducted in the United States, which does not have a single nationwide universal public healthcare system, and thus results may differ in other jurisdictions such as Canada. Moreover, previous studies are limited in generalizability (older adults or members of one United States health insurance plan only) [13, 14], or by a cross-sectional design [16]. To date, no population-based studies have assessed the effects of chiropractic utilization on medical healthcare utilization and costs within the universal public healthcare system in Canada. Addressing this knowledge gap is important to support provincial decision-making on allied healthcare policies and health services delivery for back pain.

The objective was to assess the association between chiropractic utilization and subsequent medical healthcare utilization and costs in a population-based cohort of adults with back pain within the universal public healthcare system in Ontario.

## Methods

### Study design, setting, and study period

The study design is a population-based cohort study in Ontario, with a study period from 2003 to 2018. We included four cycles of the Canadian Community Health Survey (CCHS; 2003, 2005, 2007–2008, 2009–2010), and followed the CCHS respondents up to 2018 using linked administrative data. We reported this study according to the Strengthening the Reporting of Observational Studies in Epidemiology Statement [17]. The study was conducted according to the guidelines of the Declaration of Helsinki and was approved by the Research Ethics Board at the University of Toronto (Reference #37424).

Ontario is the largest province by population (~ 14.3 million in 2018) in Canada [18]. Many medical healthcare services are publicly funded in Ontario through the government-run Ontario Health Insurance Plan, including physician visits and most basic and emergency healthcare services [19]. Chiropractic services are not paid through the Ontario Health Insurance Plan, but may be paid out-of-pocket or paid through other payer systems (extended health insurance, workers’ compensation, automobile insurance).

### Study sample and eligibility criteria

We included all Ontario respondents who self-reported back pain on at least one of four CCHS cycles (2003, 2005, 2007–2008, 2009–2010) and aged ≥ 18 years at the time of interview. Back pain was defined as self-reporting back problems diagnosed by a health professional of 6 months’ duration or greater. We excluded CCHS respondents who could not be linked to administrative data (linkage rates were 83–85%). We excluded those with a death date before the CCHS survey. CCHS respondents of multiple cycles were excluded (i.e., kept first cycle only; <1% were excluded).

### Data sources and sampling

Individual-level CCHS survey data from 2003 to 2010 were deterministically-linked to follow-up medical utilization data from health administrative databases, followed up to March 31, 2018. Deterministic linkage is an all-or-nothing linkage approach where records are matched using an exact match of unique identifying information [20]. These datasets were linked using unique encoded identifiers and analyzed at ICES (formerly known as the Institute for Clinical Evaluative Sciences). ICES is an independent, non-profit research institute whose legal status under Ontario’s health information privacy law allows it to collect and analyze healthcare and demographic data, without consent, for health system evaluation and improvement. CCHS is a cross-sectional survey administered by Statistics Canada that collects data on health determinants, outcomes, and healthcare use across Canada [21]. CCHS uses a multi-stage sampling survey design to target Canadians aged ≥ 12 years living in private dwellings and excludes persons living in institutions (e.g., long-term care), full-time members of the Canadian Forces, and persons living on-reserve and other First Nations settlements [21]. CCHS collected data from a sample of respondents every two years between 2001 and 2007. After 2007, the CCHS collected data from respondents annually. We restricted the sample to respondents aged ≥ 18 years to focus on adults with back pain. CCHS data are representative of 98% of the Canadian population aged ≥ 12 years living in private dwellings with response rates greater than 75% [21]. Detailed survey methodology is described elsewhere [22].

We used health administrative data from the Ontario Health Insurance Plan, Canadian Institute for Health Information Discharge Abstract Database and Same Day Surgeries (CIHI-DAD/SDS), and National Ambulatory Care Reporting System to capture physician billings, emergency department visits, and hospitalizations. Ontario Health Insurance Plan covers all Ontario residents, including all CCHS respondents, as a government-run universal public healthcare system. These data cover all medical providers who can claim the Ontario Health Insurance Plan and include service codes, dates of service, and associated diagnosis [23]. The CIHI-DAD/SDS collects demographic, administrative, and clinical data on hospital discharges and same-day surgeries, and the National Ambulatory Care Reporting System captures data on all hospital-based and community-based ambulatory care.

### Variables

#### Exposure – chiropractic utilization

Chiropractic utilization was defined by respondents reporting “≥1” to the CCHS question: “In the past 12 months, how many times have you seen, or talked on the telephone, about your physical, emotional or mental health with a chiropractor?” (“0” categorized as no chiropractic utilization in a specific CCHS cycle). Previous studies used this CCHS question to describe chiropractic utilization in persons with back pain [11, 24, 25].

### Outcomes – medical healthcare utilization and costs up to one- and five-year follow-up

Outcomes included cause-specific (back pain-specific) and all-cause medical utilization and healthcare costs. We assessed medical utilization and costs from the CCHS interview date up to one and five-year follow-up. Informed by literature [10, 25–28], we calculated back pain-specific visits based on billings and procedural codes, including back pain codes for physician billing, hospital visits, and spinal imaging (see Additional File – Appendix I). All-cause medical utilization included all physician visits, emergency department visits, and hospitalizations.

We calculated direct costs as total healthcare spending in Canadian dollars adjusted to 2018, using a person-centred costing approach to linked administrative databases [29]. This methodology uses an algorithm to estimate direct costs accrued by each person based on healthcare visits covered by the Ministry of Health and Long Term Care following the CCHS interview, representing the healthcare payer perspective (see Additional File – Appendix II). Comprehensive healthcare costs were available for all major sectors of healthcare spending since 2003: inpatient hospitalizations, physician visits, complex continuing care, long-term care, home services, assistive devices and pharmaceuticals [29]. Previous studies applied these methods to estimate costs for back pain and other conditions [10, 29–32].

#### Potential confounders

The following variables were considered potential confounders based on literature [33–37]:

- Sociodemographic factors (from CCHS): age, rurality, household income, education, newcomer status, ethnicity, worked in the past 12 months.
- Health-related/behavioural factors (from CCHS): smoking status, alcohol consumption, physical activity, body mass index, self-rated general health, life stress, difficulty with activities.
- Comorbidities (taken from health administrative data 2 years prior to survey date): ACG® System Collapsed Aggregated Diagnosis Groups (ADGs) using The Johns Hopkins ACG® System, version 10.0.1 (validated among adults in Ontario) [38]; health conditions using health administrative database algorithms (diabetes, hypertension, congestive heart failure, chronic obstructive pulmonary disease, dementia, stroke, coronary artery disease) [39–43].

### Data analysis

We used a survey-weighted logistic regression model that includes the aforementioned confounders and CCHS cycle to estimate a propensity-score for the probability of having chiropractic utilization compared to no chiropractic utilization. We created a propensity score-matched cohort using a nearest-neighbor 1:1-greedy matching algorithm to match participants in the exposed and unexposed groups based on logit of the propensity-score, with caliper width 0.2 times the standard deviation [44, 45]. We assessed balance of each baseline covariate between matched exposed and unexposed groups, with standardized differences 0.1 (< 10%) indicating sufficient balance [46]. After propensity-score matching, we used negative binomial regression to model the association between chiropractic utilization and rate of medical visits to compute rate ratios (RR) and 95% confidence intervals (CI). We modelled differences in costs adjusted to 2018 Canadian dollars using linear (log-transformed) models [47]. We stratified analyses by time periods to assess effects of chiropractic utilization up to one- and five-year follow-up.

All estimates incorporated CCHS survey weights and variance calculations were based on bootstrap weights with balanced repeated replication [48]. We used a pooled approach to combine CCHS cycles, which increases sample size and statistical power [49]. All costs were adjusted to 2018 Canadian dollars (CAD), and the annual exchange rate for 1 Canadian dollar was $0.77 United States Dollar in 2018 [50]. Analyses were performed using SAS version 9.4 (SAS Institute, Cary, NC) and Stata/MP 15.1 for Unix (StataCorp, College Station, TX).

### Effect modification

We assessed for effect modification by sex and prior specialist consultation using stratified analyses. Based on CCHS data, respondents reporting “≥1” consultation to a specialist in the past 12 months were categorized as having prior specialist consultation (with “0” consultations considered as no prior specialist consultation). Previous literature suggests that specialist consultation is associated with low back disability among patients in chiropractic and physiotherapy clinics [51]. Interactions with chiropractic utilization (exposure) and sex or prior specialist consultation were examined to determine if the association between chiropractic utilization and outcomes varied by sex or prior specialist consultation. Statistical tests (Wald test) were two-sided; alpha < 0.05 was considered statistically significant.

### Sensitivity analyses

We conducted sensitivity analyses to assess the potential impact of residual confounding: (1) restricting to adults aged < 65 years to address potential residual confounding by age (or other health/behavioural factors related to age); (2) restricting to adults with ≥ 1 back pain visit within 2 years prior to CCHS interview date; prior back pain-related visit(s) served as a proxy for severity of back pain requiring healthcare (potential unmeasured confounder).

### Secondary analyses

We conducted analyses with up to 15-years follow-up to assess whether observed effects were seen in the long-term. We also conducted a secondary analysis using multivariable negative binomial regression to assess the association between number of consultations with a chiropractor in the past 12 months (i.e., 0, 1–5, 6–10, > 10 consultations) and medical healthcare utilization.

## Results

There were 135,428 respondents from four CCHS cycles between 2003 and 2010 (see Additional File – Appendix III). A total of 92,175 respondents were excluded due to ineligibility (primarily age < 18 years, no self-reported back pain, or ineligible for the Ontario Health Insurance Plan), or missing exposure data (< 0.1%). Of the 29,475 respondents with back pain used for analysis, 6974 reported chiropractic utilization and 22,501 reported no chiropractic utilization. In the weighted sample, 76.1% had 0 chiropractic consultations, 7.7% had 1–5 consultations, 5.1% had 6–10 consultations, and 11.2% had more than 10 consultations in the past 12 months. After matching, there were 6972 pairs of respondents with and without chiropractic utilization.

Before matching, a lower proportion of respondents with chiropractic utilization were aged ≥ 65 years (13.9% versus 23.2%), in the lowest income quintile (8.9% versus 18.2%), and had less than secondary education (11.8% versus 21.5%), with standardized differences > 10% (Table 1). A higher proportion of respondents with chiropractic utilization worked in the past 12 months (75.6% versus 59.9%) and had excellent/very good general health (50.4% versus 39.9%). After matching, all characteristics across groups achieved standardized differences < 10%.

**Table 1:** Baseline characteristics (weighted) of adults reporting back pain with and without chiropractic utilization, propensity-score-matched cohort, CCHS 2003–2010, Ontario*

	Entire cohort				Propensity score-matched cohort			
Variable	Adults with chiropractic use n = 6974	Adults without chiropractic use n = 22,501	Absolute standardized difference	Variance ratio	Adults with chiropractic use n = 6972	Adults without chiropractic use n = 6972	Absolute standardized difference	Variance ratio
**Hard match variable**								
Female sex (%)	52.88	53.72	0.01	1.00	52.89	54.13	< 0.01	1.00
**Propensity score variables**								
**Age (%)**								
18–34 years	19.65	17.02	0.10	1.22	19.65	19.27	0.03	1.06
35–49 years	36.13	30.38	0.14	1.17	36.13	36.91	0.01	0.99
50–64 years	30.27	29.39	0.04	1.03	30.27	29.31	< 0.01	1.00
≥ 65 years	13.95	23.21	0.26	0.77	13.96	14.51	0.02	0.98
**Location of residence**								
Rural (%)	18.85	15.63	0.09	1.11	18.85	19.23	< 0.01	1.00
**Income quintile (%)**								
1 (lowest)	8.91	18.19	0.30	0.55	8.91	8.73	0.02	0.95
2	11.78	14.64	0.10	0.83	11.78	12.23	0.01	0.97
3	18.16	17.45	0.02	1.03	18.16	18.15	< 0.01	1.00
4	23.81	18.29	0.15	1.24	23.81	23.83	< 0.01	1.01
5 (highest)	26.39	18.83	0.21	1.36	26.38	26.52	0.03	1.03
Unknown	10.96	12.60	0.04	0.88	10.97	10.54	< 0.01	0.99
**Education (%)**								
Less than secondary	11.82	21.52	0.25	0.69	11.82	11.28	0.04	1.09
Secondary graduate	17.85	18.40	< 0.01	1.00	17.85	18.47	0.02	0.96
More than secondary	69.92	59.14	0.22	0.91	69.92	69.99	0.02	1.01
Unknown	0.41	0.94	0.03	0.64	0.41	0.27	0.02	1.57
**Worked in the past** **12 months (%)**								
Yes	75.61	59.94	0.39	0.87	75.61	74.46	0.06	0.96
No	19.06	29.34	0.26	0.79	19.06	19.82	0.05	0.94
Unknown	5.33	10.72	0.08	0.53	5.34	5.62	0.01	0.90
**Newcomer status (%)**								
Newcomer	21.27	30.88	0.17	0.74	21.27	21.10	0.01	1.02
Canadian-born	77.79	67.24	0.18	0.75	77.79	77.74	0.01	1.02
Unknown	0.94	1.88	0.04	0.62	0.94	1.16	< 0.01	0.98
**Ethnicity**								
White	86.49	80.96	0.12	0.71	86.49	86.07	0.01	0.96
Visible minority	12.08	16.74	0.10	0.70	12.08	12.37	0.02	0.93
Unknown	1.43	2.31	0.05	0.70	1.43	1.56	0.01	1.08
**Body mass index (%)**								
Obese, ≥ 30 kg/ m^2^	20.57	20.81	0.01	0.99	36.78	35.86	< 0.01	0.99
Overweight, 25-29.9 kg/ m^2^	36.79	34.47	0.04	1.02	20.57	20.09	0.01	1.00
Normal weight 18.5–24.9 kg/m^2^	38.22	38.23	< 0.01	1.00	38.22	38.76	0.01	1.01
Unknown	4.43	6.49	0.07	0.76	4.43	5.28	< 0.01	0.98
**Physical activity (%)**								
Active	24.21	19.08	0.09	1.14	24.20	23.28	0.01	0.99
Moderately active	24.03	22.41	0.08	1.11	24.03	24.61	0.04	1.05
Inactive	50.55	55.80	0.13	1.01	50.56	50.73	0.03	1.00
Unknown	1.21	2.72	0.08	0.53	1.21	1.39	0.01	0.90
**Alcohol consumption (%)**								
Heavy/moderate drinker	32.67	28.00	0.11	1.10	7.77	7.77	0.01	1.03
Light/never drinker	65.86	70.47	0.01	1.03	65.86	66.25	0.02	1.02
Unknown	1.47	1.53	0.11	1.16	26.36	25.98	0.02	1.02
**Smoking status (%)**								
Current smoker	21.39	26.68	0.17	0.82	21.39	21.81	0.04	0.95
Former smoker	27.68	26.93	0.02	0.98	27.68	26.49	0.17	1.06
Never smoker	47.62	42.51	0.17	1.06	47.61	48.51	0.04	1.01
Unknown	3.31	3.88	0.01	0.96	3.32	3.19	0.03	1.19
**Activity limitations**								
Often	20.41	25.71	0.20	0.81	20.41	20.78	0.06	0.92
Sometimes	28.28	27.31	0.01	1.01	28.28	27.89	0.01	0.99
Never	51.20	46.93	0.17	1.04	51.20	51.22	0.07	1.01
Unknown	0.11	0.06	0.02	1.72	0.11	0.11	< 0.01	1.14
**Life stress**								
Quite a bit/extreme stress	29.83	31.28	0.01	1.02	29.83	30.44	0.04	1.04
A bit of stress	33.01	31.18	0.01	1.01	33.01	33.00	0.03	0.98
Not at all/not very stressed	36.90	37.19	0.02	0.99	36.90	36.41	0.01	1.00
Unknown	0.26	0.36	0.02	0.64	0.26	0.15	0.01	1.23
**Self-rated general health (%)**								
Excellent/very good	50.38	39.88	0.26	1.08	50.37	51.01	0.04	1.00
Good	33.04	33.39	< 0.01	1.00	33.04	32.29	0.02	1.00
Fair/poor	16.43–16.58†	26.66	0.30	0.70	16.43–16.58†	16.55–16.70†	0.03	0.95
Unknown	0.00-0.15†	0.07	0.01	1.38	0.00-0.15†	0.00-0.15†	0.02	1.80
Chronic disease(s) (%)	37.60	44.98	0.21	0.98	37.60	37.10	0.05	0.99
**Collapsed ADGs**								
1	78.51	77.61	0.03	0.97	78.51	78.53	< 0.01	1.00
2	71.98	73.06	0.04	1.05	71.98	74.32	0.03	1.03
3	68.66	66.08	0.03	0.98	68.65	68.91	0.03	1.02
4	7.62	7.71	0.01	0.96	7.61	7.85	< 0.01	0.98
5	25.28	30.96	0.18	0.88	25.28	26.25	0.03	0.97
6	47.91	54.12	0.14	1.04	47.92	46.88	0.01	1.00
7	6.58	6.36	0.01	0.96	6.58	6.72	0.04	0.87
8	8.23	11.81	0.12	0.74	8.23	8.36	< 0.01	1.01
9	12.03	11.90	0.07	0.85	12.02	12.73	0.08	0.84
10	32.96	36.26	0.07	0.96	32.96	33.24	< 0.01	1.00
11	45.13	41.37	0.08	1.03	45.12	46.57	0.01	1.00
12	3.23	3.23	0.02	1.11	3.23	3.82	< 0.01	1.01
**CCHS cycle (%)**								
2003	24.16	23.62	0.02	1.02	24.15	24.35	< 0.01	1.00
2005	24.12	23.34	0.01	1.01	24.13	23.89	< 0.01	1.00
2007–2008	24.73	27.33	0.02	0.98	24.74	25.76	0.04	1.05
2009–2010	26.99	25.72	0.01	0.99	26.98	26.00	0.05	0.95

ADG – Aggregated Diagnosis Groups; CCHS – Canadian Community Health Survey

*Data were derived from the Ontario component of Canadian Community Health Survey (2003–2010) linked to health administrative databases. All estimates were weighted using Canadian Community Health Survey sampling weights to provide population estimates

†Ranges were used to address cell sizes < 6 to protect confidentiality

Prevalence of chiropractic utilization in the past 12 months among adults with back pain was 23.94% (95% CI 23.08–24.80) overall (Table 2), and was similar between women (23.65%, 95% CI 22.50–24.80) and men (24.26%, 95% CI 22.97–25.56).

**Table 2:** Prevalence of chiropractic utilization in adults with back pain, pooled participants of CCHS 2003–2010, Ontario*

Survey Cycle	Weighted 12-month period prevalence of chiropractic utilization, % (95% CI)
**Total Population**	N = 2,059,366
Overall	23.94% (23.08–24.80)
2003	24.35% (22.75–25.95)
2005	24.55% (22.93–26.17)
2007–2008	22.17% (20.59–23.74)
2009–2010	24.83% (22.82–26.84)

CCHS – Canadian Community Health Survey; CI - confidence interval

*Data were derived from the Ontario component of Canadian Community Health Survey (2003–2010), weighted using Canadian Community Health Survey sampling weights to provide population estimates

### Medical healthcare utilization

Among women with back pain, those with chiropractic utilization had 0.8 times lower rate of cause-specific medical visits up to 5-year follow-up than those without chiropractic utilization (RR_1year_ = 0.78, 95% CI 0.60–0.99; RR_5years_ = 0.82, 95% CI 0.68-1.00) (Table 3). There was no association between chiropractic utilization and cause-specific medical utilization for men (RR_1year_ = 1.16, 95% CI 0.76–1.78; RR_5year_ = 0.96, 95% CI 0.73–1.24).

**Table 3:** Association between chiropractic utilization and outcomes in propensity-score-matched Ontario adults with back pain*

	Up to 1-year follow-up Effect estimate, 95% CI	Up to 5-year follow-up Effect estimate, 95% CI
**Back pain-specific medical visits (number of visits per person-year)**		
Women	RR 0.78 (0.60, 0.99)	RR 0.82 (0.68, 1.00)
Men	RR 1.16 (0.76, 1.78)	RR 0.96 (0.73, 1.24)
**All-cause physician visits (number of visits per person-year)**		
Women	RR 1.00 (0.99, 1.02)	RR 0.96 (0.91, 1.03)
Men	RR 1.04 (1.01, 1.07)	RR 0.99 (0.91, 1.08)
**All-cause emergency department visits (number of visits per person-year)**		
Women	RR 1.04 (0.91, 1.19)	RR 1.03 (0.93, 1.13)
Men	RR 1.08 (0.89, 1.32)	RR 0.99 (0.88, 1.11)
**All-cause hospitalizations (number of visits per person-year)**		
Women	RR 0.91 (0.77, 1.07)	RR 0.92 (0.83, 1.02)
Men	RR 0.93 (0.74, 1.17)	RR 0.93 (0.81, 1.08)
**Healthcare costs, $CAD (adjusted to 2018)**†		
Women	1.02 (0.93, 1.13)	1.00 (0.92, 1.08)
Men	1.09 (0.96, 1.25)	0.99 (0.89, 1.10)

CAD – Canadian dollars; CI – confidence interval; RR – rate ratio

*All estimates were weighted using Canadian Community Health Survey sampling weights to provide population estimates; pooled participants with back pain from Canadian Community Health Survey cycles 2003–2010 (Ontario), followed up to 2018

†Estimates from linear (log-transformed) regression models for costs (adjusted to 2018 Canadian dollars)

There were no associations between chiropractic utilization and all-cause physician visits (RR_5years(women)_ = 0.96, 95% CI 0.91–1.03; RR_5years(men)_ = 0.99, 95% CI 0.91–1.08), all-cause hospitalizations (RR_5years(women)_ = 0.92, 95% CI 0.83–1.02; RR_5years(men)_ = 0.93, 95% CI 0.81–1.08), or all-cause emergency department visits (RR_5years(women)_ = 1.03, 95% CI 0.93–1.13; RR_5years(men)_ = 0.99, 95% CI 0.88–1.11) (Table 3).

### Costs

Healthcare costs did not differ between those with and without chiropractic utilization up to 5-year follow-up (RR_5years(women)_ = 1.00, 95% CI 0.92–1.08; RR_5years(men)_ = 0.99, 95% CI 0.89–1.10) (Table 3).

### Effect modification

Although associations between chiropractic utilization and cause-specific medical utilization varied by sex (RR_5years(women)_ = 0.82, 95% CI 0.68-1.00; RR_5year(men)_ = 0.96, 95% CI 0.73–1.24), there was no statistically significant effect modification by age (p > 0.05). There was evidence of effect modification of the association between chiropractic and cause-specific medical utilization by prior specialist consultation at 1-year (p = 0.047) but not 5-year follow-up (p > 0.05) (Table 4). Specifically, chiropractic utilization was associated with lower rates of cause-specific medical utilization at 1 year among adults without prior specialist consultation only (RR_1year(no prior consultation)_ = 0.74, 95% CI 0.57–0.97; RR_1year(prior consultation)_ = 1.17, 94% CI 0.83–1.66).

**Table 4:** Association between chiropractic utilization and outcomes in propensity-score matched adults with back pain by specialist consultation*

	Up to 1-year follow-up Effect estimate, 95% CI	Up to 5-year follow-up Effect estimate, 95% CI
**Cause-specific medical visits (number of visits per person-year)**		
Prior consultation with a specialist	RR 1.17 (0.83, 1.66)	RR 1.05 (0.87, 1.26)
No prior consultation with a specialist	RR 0.74 (0.57, 0.97)	RR 0.87 (0.72, 1.05)
**All-cause physician visits (number of visits per person-year)**		
Prior consultation with a specialist	RR 1.01 (0.99, 1.02)	RR 0.98 (0.91, 1.06)
No prior consultation with a specialist	RR 1.03 (1.01, 1.06)	RR 0.98 (0.92, 1.05)
**All-cause emergency department visits (number of visits per person-year)**		
Prior consultation with a specialist	RR 0.99 (0.82, 1.20)	RR 1.01 (0.88, 1.15)
No prior consultation with a specialist	RR 0.98 (0.85, 1.14)	RR 0.98 (0.89, 1.08)
**All-cause hospitalizations (number of visits per person-year)**		
Prior consultation with a specialist	RR 0.96 (0.80, 1.16)	RR 0.84 (0.74, 0.96)
No prior consultation with a specialist	RR 0.95 (0.77, 1.16)	RR 1.06 (0.96, 1.18)
**Healthcare costs, $CAD (adjusted to 2018)**†		
Prior consultation with a specialist	0.99 (0.87, 1.12)	0.97 (0.87, 1.08)
No prior consultation with a specialist	1.08 (0.98, 1.20)	1.01 (0.93, 1.10)

CAD – Canadian dollars; CI – confidence interval; RR – rate ratio

*All estimates were weighted using Canadian Community Health Survey sampling weights to provide population estimates; pooled participants with back pain from Canadian Community Health Survey cycles 2003–2010 (Ontario), followed up to 2018

†Estimates from linear (log-transformed) regression models for costs (adjusted to 2018 Canadian dollars)

### Sensitivity analyses

When restricting to adults aged < 65 years or with prior back pain-related visit(s), results were similar to the primary analysis overall (see Additional File – Appendix IV and V). Women with chiropractic utilization had lower rates of cause-specific medical visits (RR_5years(<65years)_ = 0.91, 95% CI 0.76–1.09; RR_5years(prior back pain visit(s))_ = 0.85, 95% CI 0.67–1.06) and there were no associations between chiropractic utilization and all-cause utilization or costs.

When assessing up to 15-year follow-up, results were similar to the primary analysis; observed effects for cause-specific utilization among women were seen in the long-term (see Additional File – Appendix VI). When assessing the association between number of chiropractic consultations and medical healthcare utilization, slightly lower rates of cause-specific healthcare utilization were observed for women with > 10 consultations (RR_5years_ = 0.85, 95% CI 0.72–1.01) and 6–10 consultations (RR_5years_ = 0.91, 95% CI 0.76–1.09) up to 5-year follow-up, but this was not statistically significant (see Additional File – Appendix VII). No associations were observed for men or all-cause medical healthcare utilization.

## Discussion

We found that among women with back pain in Ontario, those with chiropractic utilization had 0.8 times lower rates of cause-specific medical visits that those without chiropractic utilization. No associations were found between chiropractic utilization and all-cause medical healthcare utilization or costs. Chiropractic use was associated with lower rates of cause-specific medical utilization among those without previously consulting a specialist at 1-year but not 5-year follow-up, suggesting effect modification by prior specialist consultation in the short-term.

Our findings greatly extend knowledge on the effects of chiropractic use in the Ontario health system. One study reported that members of one United States health plan with back pain and chiropractic coverage had fewer surgeries and hospitalizations than those without chiropractic coverage [14]. A notable addition to the literature is our finding that women with chiropractic utilization had lower rates of cause-specific medical utilization than those without chiropractic utilization, but this association was not found in men, highlighting sex-based differences. Two studies reported that chiropractic coverage or utilization was associated with lower costs among Medicare beneficiaries or members of a United States health plan [13, 14]. In contrast, we found that chiropractic utilization was not associated with differences in healthcare costs from the perspective of the Ontario Ministry of Health and Long Term Care. This may be owing to different payer systems, with organizations such as health plan providers or Medicare as payers in the healthcare industry in the US. In Ontario, the Ministry of Health and Long Term Care perspective represents a government-run universal public health system whereby medical services are covered under the provincial health insurance plan. Chiropractic services are not covered under the Ontario Health Insurance Plan, but may be paid out-of-pocket or through other plans (e.g., extended health insurance). Our findings fill an important knowledge gap by providing new evidence on the association between chiropractic and follow-up healthcare utilization in a Canadian context, using strong approaches to account for a wide range of confounders not typically used in previous studies.

There are potential explanations for our findings. First, lower rates of cause-specific medical visits among women with chiropractic utilization may reflect differences in access to care or interprofessional referrals. In evidence-based clinical practice guidelines for the management of back pain, most non-pharmacological treatments (e.g., education, exercises, manual therapies) listed in guideline recommendations can be provided by rehabilitation professionals, including chiropractors, which may facilitate access to care [52, 53]. In contrast, there may be fewer interprofessionals referrals between medical doctors and chiropractors. A study reported that referrals between chiropractors and physicians occurred in < 10% of all encounters in chiropractic practices in Ontario [54]. Second, differences in cause-specific medical visits following chiropractic utilization by sex may reflect healthcare-seeking patterns, highlighting the importance of tailored strategies that consider gender influences. Healthcare-seeking may have biological, psychosocial, and gender-related differences, such as different pain experiences or support with activities or work [55–57].

Notably, our findings have important implications for knowledge users, including policy-makers, to support provincial decision-making on allied healthcare policies and back pain treatment strategies. Understanding the effects of chiropractic care on healthcare utilization at the health system level guides government (e.g., Ministry of Health) and health professional associations with allied healthcare delivery, resources planning, and interprofessional collaboration for the management of back pain. Our findings suggest that consultation with chiropractors among adults with back pain is not linked to additional healthcare burden or costs to the government single-payer system. Health services delivery tailored to improved access to rehabilitation providers, including chiropractors, may help address rehabilitation needs associated with back pain in Canada [58]. The World Health Organization defines rehabilitation as a set of interventions to optimize functioning when a person is experiencing limitations while interacting with their environment [59]. Common treatments provided by chiropractors include education, manual therapies, and exercise [12, 54], and rehabilitation aims to help people become independent in daily activities and participate in meaningful life roles [59]. Research exploring strategies for collaboration between chiropractors and other healthcare providers to facilitate access to evidence-based rehabilitation for adults with back pain is warranted.

### Strengths and limitations

Our study has several strengths. The CCHS is a unique source of population data on self-reported back pain, which overcomes challenges of misclassification when using back pain codes in administrative databases to ascertain back pain in the general population [25]. It also provides population-based data on chiropractic utilization using self-report, a measure not captured in administrative data within the universal public healthcare system in Canada. CCHS is representative of 98% of the community-dwelling Canadian population aged ≥ 12 years [21]. This linkage with administrative data allowed us to capture all medical encounters and direct person-level costs from the perspective of the Ontario Ministry of Health and Long Term Care. Moreover, we used rigorous methods to develop a propensity score-matched cohort to closely match adults with and without chiropractic utilization on a wide range of potential confounders, including sociodemographic, health-related, and behavioural factors.

Our study has a few limitations. First, CCHS and administrative data were only linked for those who agreed to linkage (83–85% linkage rate). However, linkage rate was high and previous analyses found adequate coverage between CCHS and administrative data [60]. We accounted for any minor differences by applying survey weights provided by Statistics Canada, which adjust for non-participation in the survey and linkage to minimize risk of selection bias. Second, there may be measurement error with self-reported chiropractic utilization. Although this CCHS question has not been assessed for validity or reliability, previous studies have used it to describe chiropractic utilization in Canada [11, 24, 25]. The wording of the CCHS question on chiropractic utilization does not allow us to determine whether back pain was the reason for seeking care. It is possible that adults with back pain reported chiropractic utilization for other comorbid health conditions. Third, data sources do not capture costs outside of the universal public healthcare system; thus, results are specific to medical utilization and costs from the healthcare payer perspective (Ontario Ministry of Health and Long Term Care) only. Fourth, data sources only capture self-reported sex and not gender, precluding any analyses to study gender-related effects. Finally, the CCHS sampling frame includes individuals living in private dwellings, and results may not be generalizable to other populations (e.g., persons living in institutions, on reserve and other First Nations settlements).

## Conclusions

We did not observe any associations between chiropractic utilization and all-cause medical healthcare utilization and costs among adults with back pain. Findings suggest sex-specific distinct patterns of back pain-specific medical healthcare utilization following chiropractic use. Specifically, sex-based differences were observed for the association between chiropractic utilization and back pain-specific healthcare visits. These findings greatly extend our knowledge to the Canadian context to guide allied health services delivery and resources planning. Given the high burden of back pain in Canada, our findings inform tailored strategies and decision-making for allied healthcare and interprofessional collaboration to strengthen effective delivery of care for back pain.

## Electronic supplementary material

Below is the link to the electronic supplementary material.

**Additional file 1:** Appendix I-VII

## Data Availability

The datasets generated and analyzed during the current study are not publicly available due to data sharing agreements and privacy policies that prohibit ICES from sharing the dataset publicly. The dataset from this study is held securely in coded form at ICES. While legal data sharing agreements between ICES and data providers (e.g., healthcare organizations and government) prohibit ICES from making the dataset publicly available, access may be granted to those who meet pre-specified criteria for confidential access, available at www.ices.on.ca/DAS (email: das@ices.on.ca). The full dataset creation plan and underlying analytic code are available from the authors upon request, understanding that the computer programs may rely upon coding templates or macros that are unique to ICES and are therefore either inaccessible or may require modification.

## Abbreviations

CCHS: Canadian Community Health Survey
CI: confidence interval
CIHI-DAD/SDS: Canadian Institute for Health Information Discharge Abstract Database and Same Day Surgeries
