# Supplementary material for: Effects of chiropractic use on medical healthcare utilization and costs in adults with back pain in Ontario, Canada from 2003 to 2018: a population-based cohort study
Source: BMC Health Serv Res. 2023 Jul 25;23:793. doi: 10.1186/s12913-023-09690-3 (PMC10367314; doi:10.1186/s12913-023-09690-3)
Supplement: Supplementary file 1 — Additional file 1: Appendix I-VII [file 12913_2023_9690_MOESM1_ESM.pdf]

SUPPLEMENTARY FILE

Title: Effects of chiropractic use on medical healthcare utilization and costs in adults with back pain in Ontario, Canada from 2003 to 2018: A population-based cohort study

Authors: Jessica J. Wong, DC, MPH, Mindy Lu, MPH, Pierre Côté, DC, PhD, Tristan Watson, MPH, Laura C. Rosella, PhD

Corresponding author: Jessica J. Wong, Epidemiology Division, Dalla Lana School of Public Health, University of Toronto, 155 College Street, 6th Floor, Toronto, Ontario M5T 3M7, Canada; email: jes.wong@mail.utoronto.ca

***

Contents

- **Appendix I.** Billing and procedural codes related to a) back pain and b) spinal imaging from health administrative data
- **Appendix II.** Detailed description of ICES costing methodology to ascertain health care costs using health administrative data
- **Appendix III.** Study flow diagram of Canadian Community Health Survey (CCHS) respondents with back pain from Ontario individually linked to provincial health administrative data from 2003 to 2010
- **Appendix IV.** Effect estimates of the association between chiropractic utilization and medical healthcare utilization and costs in propensity-score matched adults self-reporting back pain with and without chiropractic utilization, among pooled participants aged <65 years surveyed from 2003 to 2010 and followed to 2018, Canadian Community Health Survey, Ontario, Canada Community Health Survey, Ontario, Canada
- **Appendix V.** Effect estimates of the association between chiropractic utilization and medical healthcare utilization and costs in propensity-score matched adults with ≥1 back pain-related visit within 2 years from CCHS survey with and without chiropractic utilization, among pooled participants surveyed from 2003 to 2010 and followed to 2018, Canadian Community Health Survey, Ontario, Canada
- **Appendix VI.** Effect estimates of the association between chiropractic utilization and medical healthcare utilization and costs up to 15-years follow-up in propensity-score matched adults self-reporting back pain with and without chiropractic utilization, among pooled participants surveyed from 2003 to 2010 and followed to 2018, Canadian Community Health Survey, Ontario, Canada
- **Appendix VII.** Effect estimates of the association between number of chiropractic consultations and medical healthcare utilization up to 5-year follow-up in adults with self-reported back pain, among pooled participants surveyed from 2003 to 2010 and followed to 2018, Canadian Community Health Survey, Ontario, Canada

Appendix I. Billing and procedural codes related to a) back pain and b) spinal imaging from health administrative data

a) Back pain:

Type	Codes	Description
ICD-10	M4726	Other spondylosis with radiculopathy, lumbar region
	M4727	Other spondylosis with radiculopathy, lumbosacral region
	M4728	Other spondylosis with radiculopathy, sacral and sacrococcygeal region
	M4786	Other spondylosis, lumbar region
	M4787	Other spondylosis, lumbosacral region
	M4788	Other spondylosis, sacral and sacrococcygeal region
	M4796	Spondylosis, unspecified, lumbar region
	M4797	Spondylosis, unspecified, lumbosacral region
	M4798	Spondylosis, unspecified, sacral and sacrococcygeal region
	M4806	Spinal stenosis, lumbar region
	M4807	Spinal stenosis, lumbosacral region
	M4808	Spinal stenosis, sacral and sacrococcygeal region
	M4886	Other specified spondylopathies, lumbar region
	M4887	Other specified spondylopathies, lumbosacral region
	M4888	Other specified spondylopathies, sacral and sacrococcygeal region
	M4896	Spondylopathy, unspecified, lumbar region
	M4897	Spondylopathy, unspecified, lumbosacral region
	M4898	Spondylopathy, unspecified, sacral and sacrococcygeal region
	M510	Lumbar and other intervertebral disc disorders with myelopathy
	M511	Lumbar and other intervertebral disc disorders with radiculopathy
	M512	Other specified intervertebral disc displacement
	M513	Other specified intervertebral disc degeneration
	M519	Intervertebral disc disorder, unspecified
	M533	Sacrococcygeal disorders, not elsewhere classified
	M5386	Other specified dorsopathies, lumbar region
	M5387	Other specified dorsopathies, lumbosacral region
	M5388	Other specified dorsopathies, sacral and sacrococcygeal region
	M5410	Radiculopathy, multiple sites in spine
	M5416	Radiculopathy, lumbar region
	M5417	Radiculopathy, lumbosacral region
	M5418	Radiculopathy, sacral and sacrococcygeal region
	M5419	Radiculopathy, unspecified site
	M543	Sciatica
	M544	Lumbago with sciatica
	M545	Low back pain
	M548	Other dorsalgia

Type	Codes	Description
	M549	Dorsalgia, unspecified site
	M9983	Other biomechanical lesions, lumbar region
	M9993	Biomechanical lesion, unspecified, lumbar region
	M9903	Segmental and somatic dysfunction, lumbar region
	M9984	Other biomechanical lesions, sacral region
	M9994	Biomechanical lesion, unspecified, sacral region
	M9904	Segmental and somatic dysfunction, sacral region
	S335	Sprain and strain of lumbar spine
	S336	Sprain and strain of sacroiliac joint
	S337	Sprain and strain of other and unspecified parts of lumbar spine and pelvis
	S338	Sprain of other parts of lumbar spine and pelvis
	M546	Pain in thoracic spine
	M9902	Segmental and somatic dysfunction of thoracic region
	S233	Sprain of ligaments of thoracic spine, initial encounter
	M5384	Other specified dorsopathies, thoracic region
	M5385	Other specified dorsopathies, thoracolumbar region
	M6283	Muscle spasm of back
	M9908	Segmental and somatic dysfunction of rib cage
	S2341	Sprain of ribs, initial encounter
ICD-9	7213	LUMBOSACRAL SPONDYLOSIS
	72210	LUMBAR DISC DISPLACEMENT
	72252	LUMB/LUMBOSAC DISC DEGEN
	72293	DISC DIS NEC/NOS-LUMBAR
	72273	LUMB DISC DIS W MYELOPAT
	72283	POSTLAMINECT SYND-LUMBAR
	72402	SPINAL STENOSIS-LUMBAR
	7243	SCIATICA
	7242	LUMBAGO
	7245	BACKACHE NOS
	7246	DISORDERS OF SACRUM
	72470	DISORDER OF COCCYX NOS
	72471	HYPERMOBILITY OF COCCYX
	72479	DISORDER OF COCCYX NEC
	7248	OTHER BACK SYMPTOMS
	7249	BACK DISORDER NOS
	7393	SOMAT DYSFUNC LUMBAR REG
	7394	SOMAT DYSFUNC SACRAL REG
	8460	SPRAIN LUMBOSACRAL
	8461	SPRAIN SACROILIAC
	8462	SPRAIN SACROSPINATUS

Type	Codes	Description
	8463	SPRAIN SACROTUBEROUS
	8468	SPRAIN SACROILIAC NEC
	8469	SPRAIN SACROILIAC NOS
	8472	SPRAIN LUMBAR REGION
	8473	SPRAIN OF SACRUM
	8474	SPRAIN OF COCCYX
	8479	SPRAIN OF BACK NOS
	7241	Pain in thoracic spine
	7392	Nonallopathic lesions, thoracic region
	8471	Sprain of thoracic
	7398	Nonallopathic lesions, rib cage
DXCODE	724	Lumbar strain, lumbago, or sciatica
	846	Sprains and strains of sacroiliac region

ICD - International Classification of Diseases

b) Spinal imaging:

Type	Codes	Description
OHIP FEECODE	X025	Cervical spine - two or three views
	X202	Cervical spine - four or five views
	X203	Cervical spine - six or more views
	X027	Thoracic spine - two views
	X204	Thoracic spine - three or more views
	X028	Lumbar or lumbosacral spine - two or three views
	X205	Lumbar or lumbosacral spine - four or five views
	X206	Lumbar or lumbosacral spine - six or more views
	X032	Entire spine (scoliosis series) - four views
	X033	Entire spine (scoliosis series) - single view
	X031	Entire spine (scoliosis series) - two or more views
	X034	Sacrum and/or coccyx - two views
	X207	Sacrum and/or coccyx - three or more views
	X035	Sacro-iliac joints - three or more views
	X208	Sacro-iliac joints - four or more views
CCI	3SC10KM	Xray, spinal vertebrae with physically induced stress (with or without fluoroscopy)
	3SC10VA	Xray, spinal vertebrae without contrast
	3SC10VN	Xray, spinal vertebrae with fluoroscopy
	3SE10VK	Xray, intervertebral disc following intradiscal injection of contrast (with or without fluoroscopy)

	3SC12AY	Fluoroscopy, spinal vertebrae with cine/video recording
	3SC12VA	Fluoroscopy, spinal vertebrae without contrast
	3SE12VA	Fluoroscopy, intervertebral disc without contrast
	3SE12VK	Fluoroscopy, intervertebral disc following intradiscal injection of contrast
	3SF12VA	Fluoroscopy, sacrum and coccyx without contrast
	3SF12VL	Fluoroscopy, sacrum and coccyx following intraarticular injection of contrast
	3SF10VA	Xray, sacrum and coccyx without contrast
	3SF10VL	Xray, sacrum and coccyx following intraarticular injection of contrast (with or without fluoroscopy)

CCI – Canadian Classification of Health Interventions; OHIP – Ontario Health Insurance Plan

Appendix II. Detailed description of ICES costing methodology to ascertain health care costs using health administrative data[1]

Health care costs were computed using validated algorithms at ICES (formerly the Institute for Clinical Evaluative Sciences). The costing methodology computes cumulative individual-level health care costs for all publicly-funded health system encounters over time. The methodology focuses on the formal component of direct health care costs and therefore excludes copayments, costs associated with caregivers, private insurance, overheads and capital expenditures, and community-level services where an individual's health card number is not tracked.

The established costing methodology at ICES allocates health care costs to individual patients by: 1) identifying each individual's health care encounters; and 2) assigning unit costs/prices to services utilized during the encounter. Patient encounters are generally grouped into episodes and visits/claims. Costs for inpatient hospital-based episodes are computed by multiplying resource intensity weights (RIW) with cost per weighted case. RIW is a measure of the amount of hospital resources utilized during the encounter (e.g., administration, staff, supplies, drugs, technology, and equipment). For episodes such as complex continuing care, utilization measures and unit costs based on weighted days are used. The Canadian Institute for Health Information developed the methods for calculating utilization weights and unit costs for the episodes of care. For visits/claims, costs are determined at the time of utilization. These include costs for long-term care (fixed per diem costs based on government payment rates), physician costs (claims submitted to OHIP, capitation payments for primary care physicians), drug costs (costs for prescription drugs dispensed to individuals eligible for publicly funded drug coverage), home care costs (visits costs based on service type, case management and administration costs), and assistive devices (reimbursements through the Assistive Device Program).

Appendix III. Study flow diagram of Canadian Community Health Survey (CCHS) respondents with back pain from Ontario individually linked to provincial health administrative data from 2003 to 2010

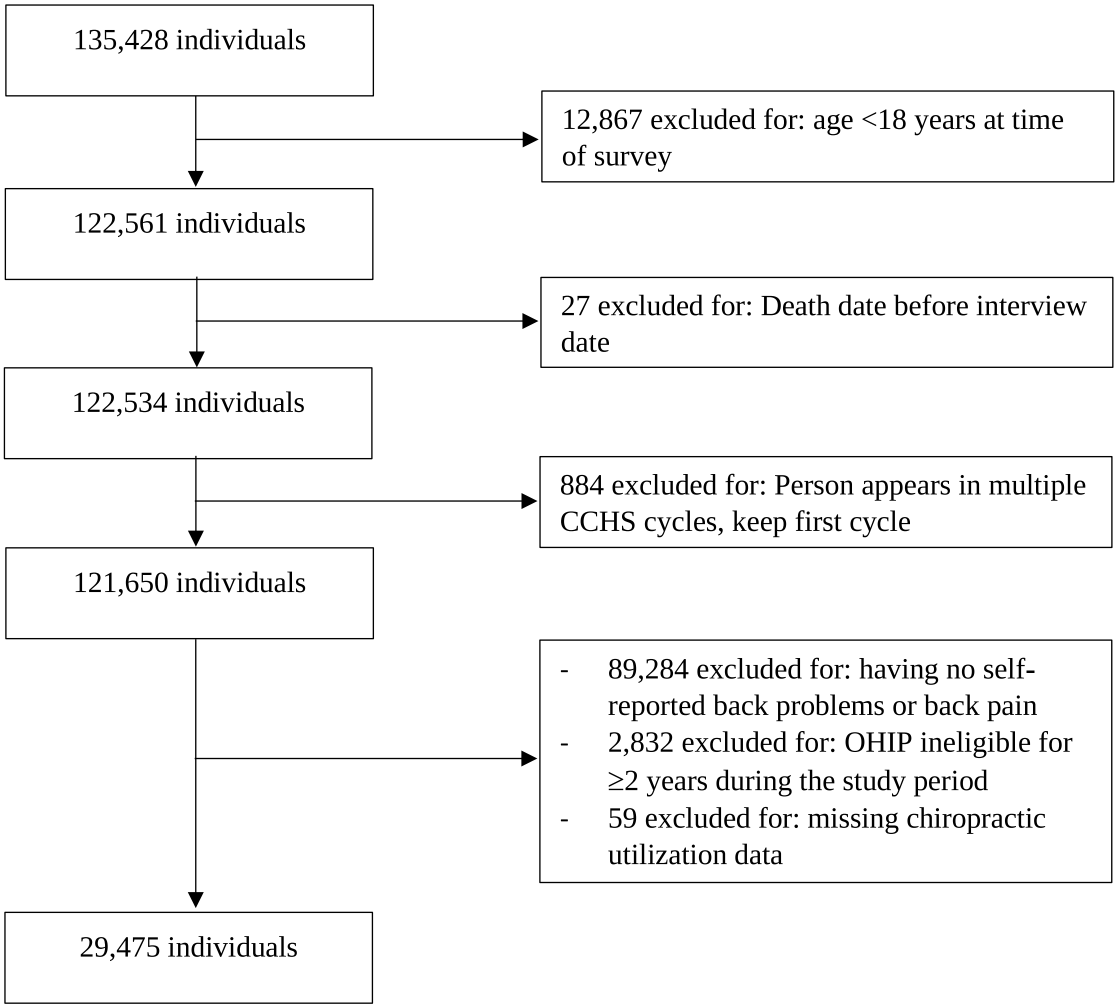

CCHS - Canadian Community Health Survey; OHIP - Ontario Health Insurance Plan

Appendix IV. Effect estimates of the association between chiropractic utilization and medical healthcare utilization and costs in propensity-score matched adults self-reporting back pain with and without chiropractic utilization, among pooled participants aged <65 years surveyed from 2003 to 2010 and followed to 2018, Canadian Community Health Survey, Ontario, Canada[a]

	**Up to 1-year follow-up Effect estimate, 95% CI**	**Up to 5-year follow-up Effect estimate, 95% CI**	**Up to 8-year follow-up Effect estimate, 95% CI**	**Up to 15-year follow-up Effect estimate, 95% CI**
**Back pain-specific medical visits (number of visits per person-year)**				
Women	RR 0.93 (0.72, 1.20)	RR 0.91 (0.76, 1.09)	RR 0.89 (0.76, 1.05)	RR 0.92 (0.78, 1.09)
Men	RR 1.21 (0.76, 1.92)	RR 1.10 (0.86, 1.41)	RR 1.09 (0.88, 1.36)	RR 1.07 (0.86, 1.33)
**All-cause physician visits (number of visits per person-year)**				
Women	RR 1.02 (0.99, 1.04)	RR 1.01 (0.94, 1.08)	RR 1.01 (0.94, 1.07)	RR 0.99 (0.93, 1.06)
Men	RR 1.04 (1.00, 1.07)	RR 0.93 (0.84, 1.04)	RR 0.94 (0.84, 1.04)	RR 0.92 (0.83, 1.02)
**All-cause emergency department visits (number of visits per person-year)**				
Women	RR 1.00 (0.84, 1.18)	RR 1.02 (0.91, 1.14)	RR 1.04 (0.93, 1.16)	1.01 (0.91, 1.13)
Men	RR 0.99 (0.78, 1.24)	RR 0.94 (0.81, 1.08)	RR 0.96 (0.84, 1.10)	0.98 (0.86, 1.12)
**All-cause hospitalizations (number of visits per person-year)**				
Women	RR 1.07 (0.87, 1.31)	RR 1.03 (0.91, 1.16)	RR 0.98 (0.89, 1.09)	RR 0.96 (0.88, 1.06)
Men	RR 0.81 (0.59, 1.10)	RR 0.83 (0.68, 1.00)	RR 0.86 (0.73, 1.01)	RR 0.85 (0.74, 0.98)
**Costs, $CAD (adjusted to 2018)[b]**				
Women	1.05 (0.94, 1.18)	1.03 (0.93, 1.13)	RR 0.98 (0.90, 1.07)	RR 0.99 (0.91, 1.08)
Men	0.96 (0.83, 1.12)	0.96 (0.84, 1.09)	RR 0.97 (0.86, 1.10)	RR 0.97 (0.86, 1.10)

CAD – Canadian dollars; CI – confidence interval; RR – rate ratio

[a]All estimates were weighted using Canadian Community Health Survey sampling weights to provide population estimates

[b]Estimates from linear (log-transformed) regression models for costs (adjusted to 2018 Canadian dollars)

Appendix V. Effect estimates of the association between chiropractic utilization and medical healthcare utilization and costs in propensity-score matched adults with ≥1 back pain-related visit within 2 years from CCHS survey with and without chiropractic utilization, among pooled participants surveyed from 2003 to 2010 and followed to 2018, Canadian Community Health Survey, Ontario, Canada[a]

	Up to 1-year follow-up Effect estimate, 95% CI	Up to 5-year follow-up Effect estimate, 95% CI	Up to 8-year follow-up Effect estimate, 95% CI	Up to 15-year follow-up Effect estimate, 95% CI
**Cause-specific medical visits (number of visits per person-year)**				
Women	RR 0.90 (0.67, 1.19)	RR 0.85 (0.67, 1.06)	RR 0.86 (0.69, 1.07)	RR 0.91 (0.73, 1.14)
Men	RR 1.22 (0.75, 1.99)	RR 1.12 (0.84, 1.51)	RR 1.14 (0.88, 1.47)	RR 1.12 (0.86, 1.46)
**All-cause physician visits (number of visits per person-year)[b]**				
Women	RR 1.00 (0.98, 1.02)	RR 0.95 (0.87, 1.05)	RR 0.98 (0.90, 1.07)	RR 0.98 (0.90, 1.07)
Men	RR 1.01 (0.97, 1.05)	RR 1.01 (0.89, 1.15)	RR 0.98 (0.87, 1.11)	RR 0.96 (0.84, 1.09)
**All-cause ED visits (number of visits per person-year)**				
Women	RR 1.01 (0.81, 1.26)	RR 1.06 (0.91, 1.24)	RR 1.04 (0.91, 1.19)	RR 1.04 (0.92, 1.19)
Men	RR 1.04 (0.76, 1.43)	RR 1.06 (0.89, 1.28)	RR 1.07 (0.90, 1.27)	RR 1.08 (0.91, 1.29)
**All-cause hospitalizations (number of visits per person-year)**				
Women	RR 0.93 (0.74, 1.18)	RR 0.91 (0.79, 1.04)	RR 0.90 (0.80, 1.02)	RR 0.91 (0.81, 1.03)
Men	RR 0.99 (0.68, 1.44)	RR 0.96 (0.77, 1.19)	RR 0.94 (0.82, 1.07)	RR 0.93 (0.78, 1.10)
**Healthcare costs, $CAD (adjusted to 2018)**				
Women	0.92 (0.77, 1.09)	0.91 (0.79, 1.04)	0.94 (0.82, 1.07)	0.94 (0.82, 1.07)
Men	1.00 (0.80, 1.26)	0.95 (0.80, 1.14)	0.99 (0.83, 1.17)	1.00 (0.84, 1.20)

CAD – Canadian dollars; CI – confidence interval; RR – rate ratio

[a]All estimates were weighted using Canadian Community Health Survey sampling weights to provide population estimates

[b]Estimates from linear (log-transformed) regression models for costs (adjusted to 2018 Canadian dollars)

Appendix VI. Effect estimates of the association between chiropractic utilization and medical healthcare utilization and costs up to 15-year follow-up in propensity-score matched adults self-reporting back pain with and without chiropractic utilization, among pooled participants surveyed from 2003 to 2010 and followed to 2018, Canadian Community Health Survey, Ontario, Canada Community Health Survey, Ontario, Canada[a]

	**Up to 1-year follow-up Effect estimate, 95% CI**	**Up to 5-year follow-up Effect estimate, 95% CI**	**Up to 8-year follow-up Effect estimate, 95% CI**	**Up to 15-year follow-up Effect estimate, 95% CI**
**Cause-specific medical visits (number of visits per person-year)**				
Women	RR 0.78 (0.60, 0.99)	RR 0.82 (0.68, 1.00)	RR 0.83 (0.69, 1.01)	RR 0.83 (0.68, 1.01)
Men	RR 1.16 (0.76, 1.78)	RR 0.96 (0.73, 1.24)	RR 0.99 (0.80, 1.22)	RR 1.00 (0.80, 1.24)
**All-cause physician visits (number of visits per person-year)[b]**				
Women	RR 1.00 (0.99, 1.02)	RR 0.96 (0.91, 1.03)	RR 0.97 (0.92, 1.03)	RR 0.96 (0.91, 1.02)
Men	RR 1.04 (1.01, 1.07)	RR 0.99 (0.91, 1.08)	RR 1.00 (0.92, 1.09)	RR 0.97 (0.89, 1.06)
**All-cause ED visits (number of visits per person-year)**				
Women	RR 1.04 (0.91, 1.19)	RR 1.03 (0.93, 1.13)	RR 1.03 (0.94, 1.14)	RR 1.02 (0.94, 1.11)
Men	RR 1.08 (0.89, 1.32)	RR 0.99 (0.88, 1.11)	RR 1.01 (0.91, 1.13)	RR 1.01 (0.91, 1.12)
**All-cause hospitalizations (number of visits per person-year)**				
Women	RR 0.91 (0.77, 1.07)	RR 0.92 (0.83, 1.02)	RR 0.90 (0.82, 0.99)	RR 0.91 (0.84, 0.99)
Men	RR 0.93 (0.74, 1.17)	RR 0.93 (0.81, 1.08)	RR 0.95 (0.83, 1.07)	RR 0.92 (0.82, 1.03)
**Healthcare costs, $CAD (adjusted to 2018)**				
Women	1.02 (0.93, 1.13)	1.00 (0.92, 1.08)	0.98 (0.90, 1.04)	0.97 (0.90, 1.04)
Men	1.09 (0.96, 1.25)	0.99 (0.89, 1.10)	1.02 (0.91, 1.13)	1.01 (0.91, 1.13)

CAD – Canadian dollars; CI – confidence interval; RR – rate ratio

[a]All estimates were weighted using Canadian Community Health Survey sampling weights to provide population estimates

[b]Estimates from linear (log-transformed) regression models for costs (adjusted to 2018 Canadian dollars)

Appendix VII. Effect estimates of the association between number of chiropractic consultations and medical healthcare utilization up to 5-year follow-up in adults with self-reported back pain, among pooled participants surveyed from 2003 to 2010 and followed to 2018, Canadian Community Health Survey, Ontario, Canada[a]

	**Up to 1-year follow-up Effect estimate, 95% CI**		**Up to 5-year follow-up Effect estimate, 95% CI**	
	**Unadjusted**	**Adjusted[b]**	**Unadjusted**	**Adjusted[b]**
**Cause-specific medical visits (number of visits per person-year)**	**RR (95% CI)**	**RR (95% CI)**	**RR (95% CI)**	**RR (95% CI)**
**Women**				
0 chiropractic consultations	Reference - 1.00	Reference - 1.00	Reference - 1.00	Reference - 1.00
1-5 chiropractic consultations	0.90 (0.68-1.19)	0.98 (0.75-1.28)	0.89 (0.72-1.09)	0.98 (0.81-1.19)
6-10 chiropractic consultations	0.85 (0.61-1.19)	1.06 (0.72-1.57)	0.75 (0.62-0.91)	0.91 (0.76-1.09)
>10 chiropractic consultations	0.83 (0.61-1.13)	0.86 (0.67-1.09)	0.74 (0.60-0.93)	0.85 (0.72-1.01)
**Men**				
0 chiropractic consultations	Reference - 1.00	Reference - 1.00	Reference - 1.00	Reference - 1.00
1-5 chiropractic consultations	1.34 (0.62-2.87)	1.07 (0.73-1.56)	1.15 (0.81-1.65)	1.15 (0.99-1.45)
6-10 chiropractic consultations	0.65 (0.44-0.96)	0.70 (0.47-1.03)	0.85 (0.64-1.14)	0.98 (0.75-1.26)
>10 chiropractic consultations	0.65 (0.57-0.73)	0.99 (0.75-1.31)	0.70 (0.49-1.08)	1.00 (0.80-1.25)
**All-cause physician visits (number of visits per person-year)**				
**Women**				
0 chiropractic consultations	Reference - 1.00	Reference - 1.00	Reference - 1.00	Reference - 1.00
1-5 chiropractic consultations	0.99 (0.96-1.01)	1.00 (0.97-1.02)	0.86 (0.78-0.94)	1.01 (0.93-1.09)
6-10 chiropractic consultations	1.02 (1.00-1.04)	1.03 (1.01-1.05)	0.81 (0.74-0.87)	0.96 (0.89-1.03
>10 chiropractic consultations	1.01 (0.99-1.02)	1.02 (1.00-1.04)	0.79 (0.74-0.83)	0.97 (0.92-1.02)
**Men**				
0 chiropractic consultations	Reference - 1.00	Reference - 1.00	Reference - 1.00	Reference - 1.00
1-5 chiropractic consultations	1.03 (1.01-1.07)	1.05 (1.03-1.08)	0.92 (0.83-1.02)	1.13 (1.03-1.24)
6-10 chiropractic consultations	1.01 (0.97-1.05)	1.03 (0.99-1.07)	0.92 (0.80-1.06)	1.11 (0.97-1.26)
>10 chiropractic consultations	1.04 (1.01-1.08)	1.06 (1.03-1.10)	0.81 (0.73-0.90)	0.98 (0.90-1.07)

	Up to 1-year follow-up<br>Effect estimate, 95% CI		Up to 5-year follow-up<br>Effect estimate, 95% CI	
	**Unadjusted**	**Adjusted[b]**	**Unadjusted**	**Adjusted[b]**
**All-cause emergency department visits (number of visits per person-year)**				
**Women**				
0 chiropractic consultations	Reference - 1.00	Reference - 1.00	Reference - 1.00	Reference - 1.00
1-5 chiropractic consultations	0.90 (0.74-1.10)	1.01 (0.83-1.22)	0.94 (0.81-1.09)	1.12 (0.98-1.28)
6-10 chiropractic consultations	0.93 (0.75-1.16)	1.07 (0.89-1.29)	0.85 (0.73-0.99)	1.02 (0.89-1.16)
>10 chiropractic consultations	0.84 (0.72-0.98)	1.00 (0.85-1.17)	0.81 (0.72-0.92)	0.99 (0.88-1.12)
**Men**				
0 chiropractic consultations	Reference - 1.00	Reference - 1.00	Reference - 1.00	Reference - 1.00
1-5 chiropractic consultations	1.29 (1.01-1.66)	1.51 (1.20-1.90)	0.93 (0.80-1.08)	1.11 (0.96-1.28)
6-10 chiropractic consultations	0.58 (0.43-0.79)	0.67 (0.52-0.87)	0.83 (0.69-0.98)	1.00 (0.86-1.19)
>10 chiropractic consultations	0.67 (0.55-0.80)	0.82 (0.67-1.00)	0.77 (0.67-0.89)	0.92 (0.80-1.05)
**All-cause hospitalizations (number of visits per person-year)**				
**Women**				
0 chiropractic consultations	Reference - 1.00	Reference - 1.00	Reference - 1.00	Reference - 1.00
1-5 chiropractic consultations	0.88 (0.68-1.12)	1.11 (0.87-1.43)	0.78 (0.69-0.88)	0.96 (0.85-1.09)
6-10 chiropractic consultations	0.74 (0.60-0.91)	0.94 (0.75-1.16)	0.83 (0.73-0.94)	1.01 (0.90-1.14)
>10 chiropractic consultations	0.82 (0.70-0.96)	1.02 (0.86-1.21)	0.82 (0.75-0.91)	1.03 (0.93-1.15)
**Men**				
0 chiropractic consultations	Reference - 1.00	Reference - 1.00	Reference - 1.00	Reference - 1.00
1-5 chiropractic consultations	0.79 (0.61-1.01)	1.00 (0.78-1.28)	0.79 (0.68-0.92)	1.04 (0.88-1.23)
6-10 chiropractic consultations	0.68 (0.52-0.89)	0.80 (0.61-1.05)	0.90 (0.77-1.05)	1.16 (0.97-1.38)
>10 chiropractic consultations	0.98 (0.70-1.35)	1.18 (0.88-1.58)	0.78 (0.62-0.97)	0.99 (0.78-1.26)

CI – confidence interval; RR – rate ratio

[a]All estimates were weighted using Canadian Community Health Survey sampling weights to provide population estimates

[b]Adjusted for age, rurality, household income, education, immigrant status, ethnicity, work status, smoking, alcohol, physical activity, body mass index, self-rated general health, life stress, difficulty with activities, and comorbidities

Reference for Supplementary File

1. Wodchis W, Bushmeneva K, Nikitovic M, McKillop I. Guidelines on Person level Costing Using Administrative Databases in Ontario. Toronto: Health System Performance Research Network; 2013.
